# CFD Convective Flow Simulation of the Varying Properties of CO_2_-H_2_O Mixtures in Geothermal Systems

**DOI:** 10.1155/2015/843068

**Published:** 2015-03-23

**Authors:** S. Yousefi, A. D. Atrens, E. Sauret, M. Dahari, K. Hooman

**Affiliations:** ^1^Mechanical Engineering Department, Islamic Azad University, Gachsaran Branch, Gachsaran, Iran; ^2^School of Mechanical and Mining Engineering, The University of Queensland, Brisbane, QLD 4072, Australia; ^3^School of Chemistry, Physics and Mechanical Engineering, Queensland University of Technology, Brisbane, QLD 4000, Australia; ^4^Department of Mechanical Engineering, Faculty of Engineering, University of Malaya, 50603 Kuala Lumpur, Malaysia

## Abstract

Numerical simulation of a geothermal reservoir, modelled as a bottom-heated square box, filled with water-CO_2_ mixture is presented in this work. Furthermore, results for two limiting cases of a reservoir filled with either pure water or CO_2_ are presented. Effects of different parameters including CO_2_ concentration as well as reservoir pressure and temperature on the overall performance of the system are investigated. It has been noted that, with a fixed reservoir pressure and temperature, any increase in CO_2_ concentration leads to better performance, that is, stronger convection and higher heat transfer rates. With a fixed CO_2_ concentration, however, the reservoir pressure and temperature can significantly affect the overall heat transfer and flow rate from the reservoir. Details of such variations are documented and discussed in the present paper.

## 1. Introduction

Simultaneous power generation and geosequestration make CO_2_ a very attractive choice for geothermal power plants. As such, carbon-dioxide-based engineered geothermal systems (CO_2_-EGS) have been previously proposed as an alternative to water-based EGS systems [1]. Subsequent studies added further details and reported possibility of improved energy extraction [2–7]. Interestingly, the “dry-out period,” or transition from an initially water-filled EGS system to a CO_2_-rich one [8–11] has received a lot of attention mainly because of possible mineral dissolution and precipitation as a result of changes in the reservoir fluid composition and reservoir permeability alteration [12–14]. Additionally, [15] investigated the effects of CO_2_-rich phase compositions on the production flow rate and the heat extraction from the reservoir. What is yet to be reported in the literature is a detailed numerical simulation of a water-CO_2_ mixture filling a reservoir. Simple thermodynamic analysis of a reservoir shows that more heat can be extracted (compared to a water-saturated reservoir) mainly because a CO_2_-water mixture is more buoyant than pure water. This could significantly affect the energy extraction from a water-saturated reservoir. Most of these reservoirs suffer from permeability drops with depths. As such, having a more buoyant fluid which could move up against gravity, at least partially, not only is desirable but also is going to significantly affect the drilling cost which is expected to grow exponentially with the well depth [16–18].

The role of fluid migration in the build-up of heat in underground geothermal systems is not well understood. It is known that degassing of CO_2_ with isotopic composition indicating mantle-sources in regions of tectonic activity is associated with locally elevated geothermal temperatures [19–21]. Convective fluid plumes may play a role in enhancing heat flows from the mantle to geothermal reservoirs and within the reservoirs themselves. Additionally, convection within a geothermal reservoir may enhance the productive life-time of geothermal reservoir systems by enhancing heat supply from underlying strata and by ensuring a more even distribution of thermal energy throughout the reservoir, that is, by off-setting localized cooling along major flow paths.

This could also imply that for a water-saturated reservoir which is classified as nonproductive, mainly due to lack of convection currents, injection of CO_2_ can lead to formation of convection cells and thus facilitate heat extraction. In what follows, a numerical analysis of this problem is presented to quantitatively measure the improved convective flow patterns and enhanced heat transfer from the reservoir. This is intended to provide insight as to the possible mechanisms by which CO_2_ presence in or addition to underground reservoirs could lead to enhancement of convective heat transfer.

## 2. Modelling

**Figure 1:**
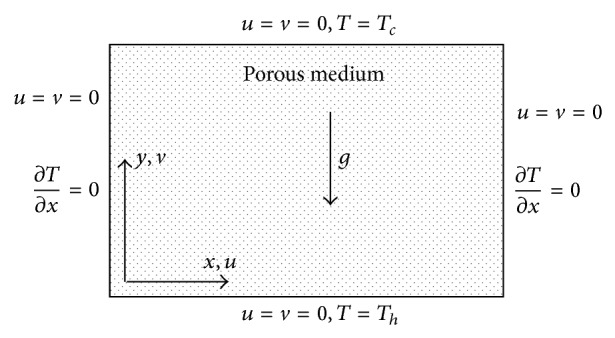
Schematic view of the computational domain.

The reservoir is modelled as a bottom-heated square box with adiabatic lateral boundaries and a cold top wall as Figure 1 shows. The cold and hot temperatures are varied from 331 to 431 K and 416 to 516 K, respectively, in a way that the hot-cold temperature difference remains at 85 K for each case. For constant properties, and of course with the same reservoir size, porosity, and permeability, one would expect the results to be the same as long as the temperature difference is not altered. It will, however, be shown in the forthcoming sections that this is not the case in our problem as properties significantly vary with both temperature and pressure. The reservoir porosity is fixed at 0.05 and the permeability-length product is kept constant at 10^−11^ m^3^ similar to Haghshenas Fard et al. [7] with no through-flow. The reservoir pressure is varied from 20 to 60 MPa (equivalent to the hydrostatic pressure of approximately 2 to 6 km of water) to cover a wide range of practical applications for geothermal development. The flow is modelled using Darcy flow model with the single-phase fluid properties obtained as linear superposition of those of individual fluids weighted with their respective fraction; that is,

(1) $$\begin{array}{c} \rho_{g}=\omega_{\gamma }\rho_{\gamma }+\omega_{\varepsilon }\rho_{\varepsilon },\\ \beta_{g}=\omega_{\gamma }\beta_{\gamma }+\omega_{\varepsilon }\beta_{\varepsilon },\\ c_{pg}=\omega_{\gamma }{c_{p\gamma }}_{}+\omega_{\varepsilon }{c_{p\varepsilon }}_{},\\ \mu_{g}=\omega_{\gamma }\mu_{\gamma }+\omega_{\varepsilon }\mu_{\varepsilon }. \end{array}$$

The real (i.e., nonideal) properties are determined for pure CO_2_ using a Helmholtz-free-energy based equation of state [22] and for pure H_2_O using the International Association for the Properties of Water and Steam equation of state [23]. The use of a single-phase basis for modelling is to enable examination of the effect of changing properties without additional complexities of two-phase flow separation behaviour. Further comment is provided in the discussion. The reservoir properties are then obtained similarly using the porosity and solidity as the weight-functions as follows:

(2) $$\begin{array}{c} \lambda_{R}=\phi \left(\omega_{\gamma }\lambda_{\gamma }+\omega_{\varepsilon }\lambda_{\varepsilon }\right)+\left(1-\phi \right)\lambda_{m}. \end{array}$$

One notes that in the above formulation changes in the reservoir porosity and permeability are not taken into account while fluid properties are updated in each iteration.

An in-house code, used by Hooman and Gurgenci [24], was cross-validated with the commercially available software ANSYS-FLUENT and used to create and mesh the geometry and finally solve the governing equations. The governing equations were derived as for standard two-dimensional heat transfer conditions, based on the assumptions of adiabatic lateral walls and constant temperature for the top and bottom boundaries, and are as follows:

(3) $$\begin{array}{c} \frac{\partial u}{\partial x}+\frac{\partial v}{\partial y}=0,\\ \frac{\partial u}{\partial y}-\frac{\partial v}{\partial x}=-\frac{\rho g\beta K}{\mu }\frac{\partial T}{\partial x},\\ u\frac{\partial T}{\partial x}+v\frac{\partial T}{\partial y}=\alpha \left(\frac{\partial^{2}T}{\partial x^{2}}+\frac{\partial^{2}T}{\partial y^{2}}\right), \end{array}$$

subject to the boundary conditions illustrated in Figure 1.

## 3. Numerical Details

**Figure 2:**
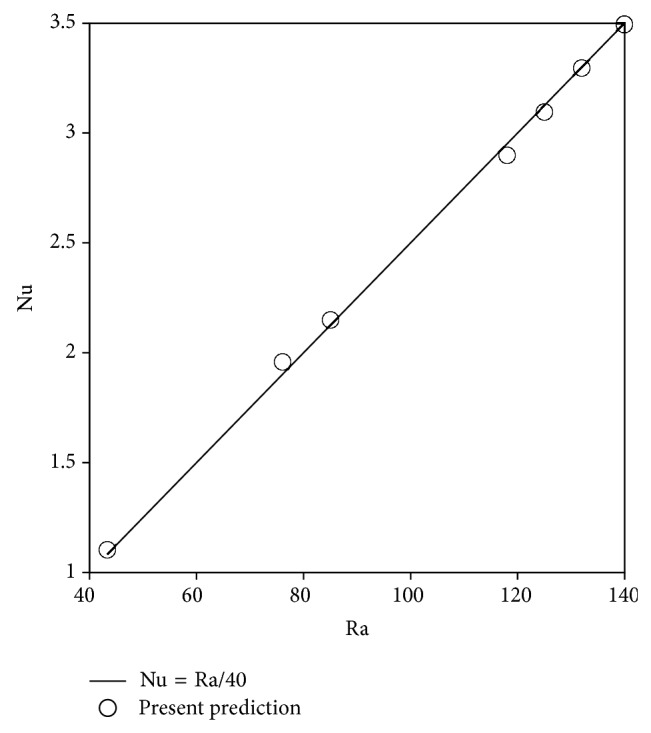
Validation of present CFD results against existing correlation for pure water.

Grid independence was verified by running the software on different combination of grid sizes. It was observed that the results changed less than 2% when a 100 × 100 mesh system is used instead of a finer mesh with 200 × 200 grid points. Results are also verified for constant property free convection of water in a porous cavity, that is, the Darcy-Benard problem. It was noted that the correlation between Nusselt number (Nu) and Rayleigh number (Ra) Nu = Ra/40 best fits out numerical data, as Figure 2 shows. Ra in this instance is determined as per

(4) $$\begin{array}{c} \text{Ra}=\frac{g\beta \rho }{\mu \alpha }\left(T_{h}-T_{c}\right)KH. \end{array}$$

As a further check on the accuracy of our results, variable property Darcy-Benard free convection of pure water in a porous cavity was investigated to observe that using the reference temperature approach of Hooman and Gurgenci [24] the above correlation can still be used within 5%.

## 4. Results and Discussion

**Figure 3:**
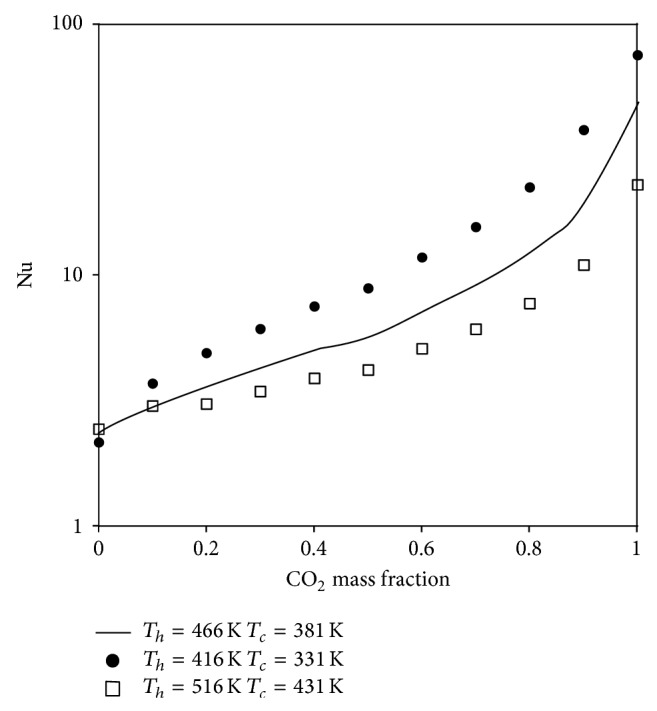
Nusselt number versus CO_2_ mass fraction for different *T*_*h*_ and *T*_*c*_ combinations with Δ*T* = 85 K.

In what follows we focus on free convection heat and fluid flow of a water-CO_2_ mixture in a porous cavity. We use Nu and maximum flow rate as our metrics to evaluate the strength of convective flow patterns. Nu is the total heat transfer divided by that of pure conduction through the same cavity (no convective flow patterns). As such, any Nu value in excess of unity shows some degree of convection. Obviously, higher Nu values mark stronger convective cells. The flow rate reported here is the one induced by free convection only, that is, without a well-head pump or any other suction/injection mechanisms. We systematically change the CO_2_ mass fraction from zero (pure water) to unity (pure CO_2_) over a range of reservoir pressure and temperature in a way that the hot-cold temperature difference remains the same. For a constant property subcritical fluid flow, one would expect that, with the same temperature difference and, hence, the same Rayleigh number (Ra), the overall heat transfer and fluid flow will not alter. However, as CO_2_ is supercritical within the range of conditions of underground reservoir systems, that is not the case for mixtures of CO_2_ and H_2_O, as demonstrated by Figure 3. This figure shows Nu versus CO_2_ mass fraction at 20 MPa with the same hot-cold temperature difference but with different hot and cold temperatures as denoted on the plots. As seen, the heat transfer increases with CO_2_ mass fraction for any given *T*_*h*_ and *T*_*c*_ combination. Furthermore, moving from pure water to pure CO_2_, the increase in heat transfer is significant; about one order of magnitude is the minimal heat transfer augmentation. More interestingly, however, is the fact that Nu is the highest with the lowest *T*_*c*_ (and obviously lowest *T*_*h*_ to maintain the same Δ*T* of 85 K) mainly because the lower temperatures are closer to those of pseudocritical conditions where Ra is expected to reach a maximum value; see also Forooghi et al. [25–28]. This is obviously in favour of low temperature geothermal reservoirs which may not be productive when pure water is the working fluid.

**Figure 4:**
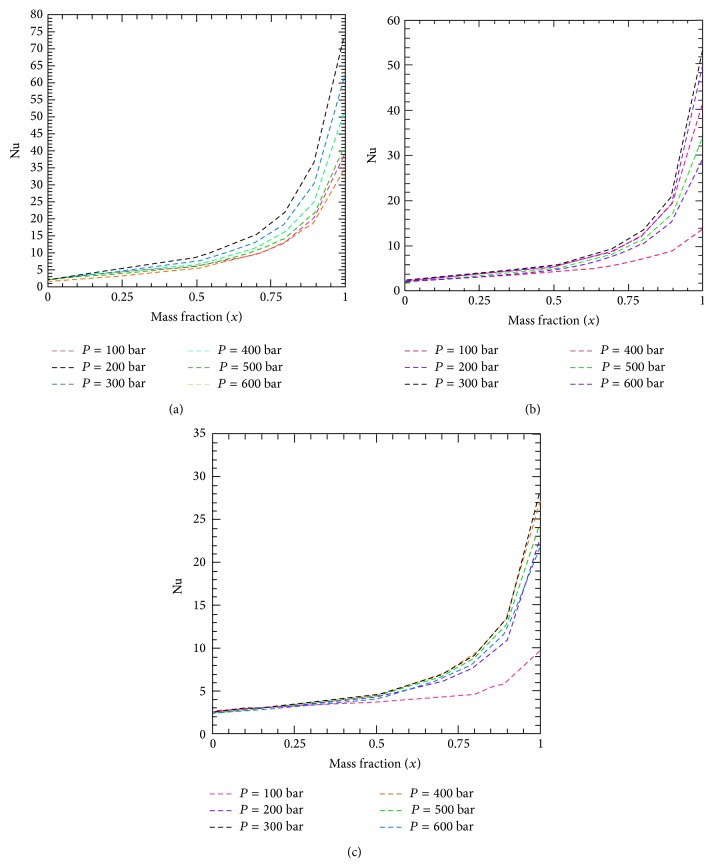
Nusselt number versus CO_2_ mass fraction for different reservoir pressures and temperature combinations: (a) *T*_*h*_ = 416 K; *T*_*c*_ = 331 K, (b) *T*_*h*_ = 466 K; *T*_*c*_ = 381 K, and (c) *T*_*h*_ = 516 K; *T*_*c*_ = 431 K.

Figures 4(a)–4(c) are presented to demonstrate Nu versus CO_2_ mass fraction for different reservoir pressures and hot-cold temperature combinations. Nu increases with mass fraction for a fixed pressure and hot-cold temperature conbination. Comparing the relationship between any of Figures 4(a)–4(c) for a fixed pressure will result in conclusions similar to what were drawn based on close examination of Figure 3. That is, heat transfer increases for temperatures close to pseudocritical conditions. Moreover, based on plots in the same chart, increasing the pressure leads to lower heat trasnfer rates for a fixed CO_2_ mass fraction and temperature. This could be explained as the obvious decrease in compressibility and increase in the fluid density with higher pressures, with a fixed fluid temperature, which will lead to lower thermal expansion coefficients. As a result, at the same temperature, either of the two fluids will be less buoyant at higher pressures when compared to lower ones, so will be the mixture in the absence of any phase transitions.

**Figure 5:**
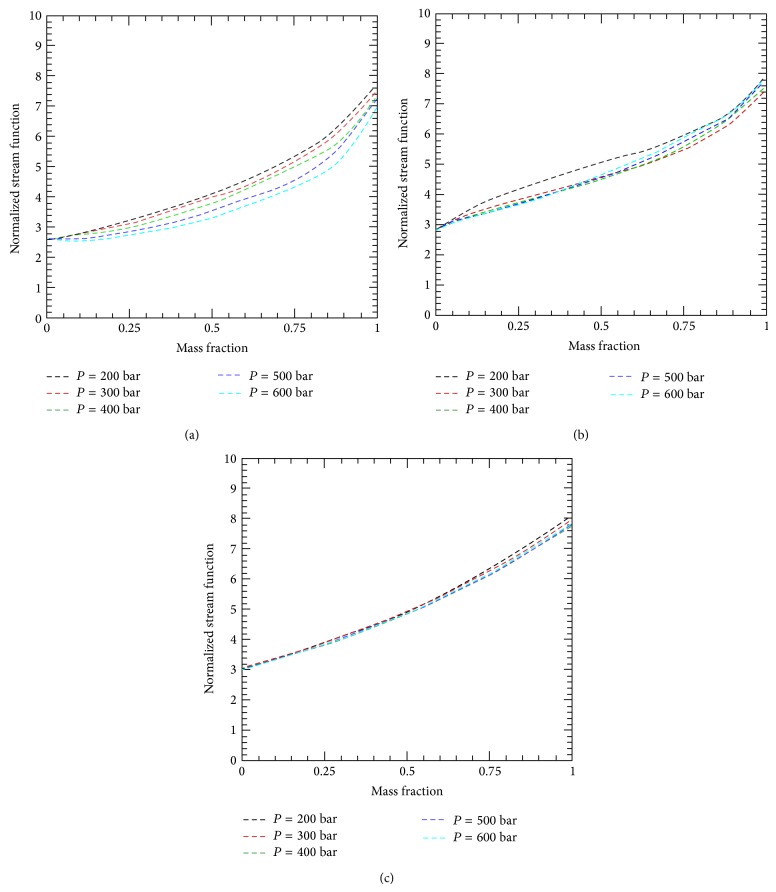
Dimensionless mass flow rate versus CO_2_ mass fraction for different pressures and temperature combinations: (a) *T*_*h*_ = 416 K; *T*_*c*_ = 331 K, (b) *T*_*h*_ = 466 K; *T*_*c*_ = 381 K, and (c) *T*_*h*_ = 516 K; *T*_*c*_ = 431 K.

The convective flow rates also reflect a dependence on compressibility, as demonstrated by Figure 5. The dimensionless flow rate (normalized stream function on the vertical axis) is obtained by normalizing the actual flow rate with appropriate scales for velocity, area, and density:

(5) $$\begin{array}{c} \xi =\frac{\dot{m}}{\left(\rho uA\right)}. \end{array}$$

Mathematically, it means that we used the group *ρAu* to nondimensionalize the flow rate. It needs to be mentioned that the choice of these parameters is optional but we tried to use constant values for density and length to make it easy for the reader to generate estimates, based on our calculation, for expected flow rates through a given reservoir. Moreover, what we are more interested in is the trend of the flow rate plot against the mass fraction than the actual flow rate values. In doing so, the (constant) density of water at atmospheric condition is used where the unit area is used defined as the length of the cavity multiplied by unity (1 m). The flow velocity, for single-phase constant property case, is assumed to be linearly proportional to the product of the thermal diffusivity and Ra^1/2^ and inverse linearly proportional to the cavity length; for example,

(6) $$\begin{array}{c} u\sim \frac{\alpha }{H}\sqrt{\text{Ra}}. \end{array}$$

The flow rate is given by

(7) $$\begin{array}{c} \dot{m}=\rho Hu\sim \rho \alpha \sqrt{\text{Ra}}. \end{array}$$

The product of thermal diffusivity and density is independent of density and leaves us with a group *λ*/*c*_*p*_. Consequently the mass flow rate scale is represented as

(8) $$\begin{array}{c} \dot{m}=\rho Hu\sim \frac{k}{c_{p}}\sqrt{\text{Ra}}. \end{array}$$

While *λ* and *c*_*p*_ are calculated under standard atmospheric conditions, Ra is affected by fluid property variation and following the use of (1)-(2). This flow rate here is the buoyancy-induced flow rate due to changes in fluid density. The buoyancy-induced flow leads to an upward movement of hot fluid toward the top wall, where it is cooled and then displaced by other rising hot fluid. Results of mass flow rate normalized by using (8) above are presented in Figure 5 for different mass fractions, pressure, and temperature combinations. Similar to Nu plots, one notes that the mass flow rate is sensitive not only to the temperature difference but also to the actual wall temperature values. Furthermore, higher CO_2_ mass fraction leads to higher flow rates. It can be noted, moving from Figures 5(a) to 5(c), that flow rates are less sensitive to pressure as *T*_*h*_ is increased. With a fixed *T*_*h*_ and *T*_*c*_, one notes different trends in flow rate when pressure changes. Depending on the temperature values, an increase in pressure can either increase (Figure 5(a)) or decrease the flow rate (Figure 5(b)).

This work is an initial analysis of the role of CO_2_ enhancement of convective heat transfer within geothermal reservoirs. It deliberately assesses the behaviour of a single-phase mixture of the two components. Further work is necessary to extend this to account for multiple phases. There are three particular qualitative effects through which multiphase flow is expected to alter the results presented here.

1. Transient exsolution of dissolved CO_2_ as bubbles within the two-phase region should lead to local enhancement of convective flow around the bubble due to its upwards buoyancy-driven motion. One expectation of this would be an increase in the gradient of convective heat flux with mass fraction (i.e., *dξ*/*dx*) at the bubble line, where CO_2_ begins to exsolve from the H_2_O phase.
2. Relative permeability within the two-phase region would act to reduce enhancement of flows, as the reservoir permeability to the minor phase within a two-phase flow is typically substantially reduced.
3. Under steady-state conditions, there would be an expectation of phase separation into two horizontal phases based on relative density, that is, an upper CO_2_-rich phase and a lower H_2_O-rich phase. The upper phase would experience significantly enhanced convective heat transfer rates, as it would have internal heat transfer characteristics similar to the right sides of Figures 4 and 5. Additionally, the heat transfer would be further enhanced by the temperature-dependent solubility of H_2_O in the CO_2_-rich phase, leading to additional H_2_O evaporating into the CO_2_ phase at the boundary between the two phases and condensing at the upper surface of the reservoir. The H_2_O-rich phase would experience the converse effect, depressing the rate of heat transfer, although CO_2_ solubility in H_2_O is far less dependent on temperature than that of H_2_O in CO_2_ [11].

These expected qualitative behaviours require further analysis accounting for multiphase flow behaviour to determine their relative contribution to overall convective heat flow enhancement.

However, the sum of these changes is not expected to reverse the overall trend demonstrated here, of increased convective heat flux as CO_2_ is added to the reservoir system. Considering that the results presented here indicate that CO_2_ may enhance flow rates by up to a factor of 2.67, we conclude that this is a potentially important mode of heat transport within geothermal reservoirs and warrants further study. We anticipate the next steps to be consideration of the additional flow behaviours when multiple phases are present.

## 5. Conclusion

The effect on convective heat transport within a closed reservoir system of varying fluid composition was analysed by CFD modelling of a single-phase fluid with properties derived from a composition-dependent average of pure CO_2_ and pure H_2_O. As compositional properties were varied from H_2_O toward that of CO_2_, substantial increases were observed in Nusselt number (by a factor of up to 10) and normalised stream function (by a factor of up to 2.67). We conclude that this indicates substantial increase in convective heat transport.

Convective heat transport may be further modified by multiphase flow behaviours, and we conclude that, due to the potential magnitude of heat flow enhancement by the addition of CO_2_, further research exploring the effect of these behaviours on heat transport is warranted.

We anticipate this finding to have implications for the study of natural geothermal reservoirs, where the role of dissolved gas exsolution on heat transfer enhancement remains unquantified. Additionally, these findings may be of potential interest with regard to CO_2_ injection into geothermal reservoirs, as it may lead to improved productivity through the mechanisms elucidated in this work.

The magnitude of this effect on the thermal productivity of geothermal power plants is difficult at this stage to quantify and probably not meaningful to speculate on due to the limitation of assessing only single-phase flow behaviours within the reservoir. These findings do demonstrate, however, that reservoirs with elevated CO_2_ content will experience greater convective heat transfer and therefore be of comparatively higher temperature (and therefore of greater resource value). Additionally, it can be concluded that any increase in CO_2_ content of an existing reservoir will enhance convective flow behaviours (although the true magnitude of this effect will depend on two-phase flow behaviour as well as the particulars of the reservoir) and consequently will enhance the productivity and/or the longevity of geothermal energy extraction.

## Glossary

*ρ*: Density, kg m^−3^
*ω*: Mass fraction, dimensionless
*β*: Thermal expansion coefficient, K^−1^
*μ*: Dynamic viscosity, Pa s
*λ*: Thermal conductivity W m^−1^ K^−1^
*ϕ*: Reservoir porosity, dimensionless
*c*_*p*_: constant-pressure heat capacity, kJ kg^−1^ K^−1^
*g*: Gravitational acceleration, 9.81 m s^−2^
*u*: Horizontal component of fluid velocity, m s^−1^
*v*: Vertical component of fluid velocity, m s^−1^
Nu: Nusselt number, dimensionless
Ra: Rayleigh number, dimensionless
*T*: Temperature, K
*ξ*: Normalised flow rate, dimensionless
*m*: Mass flow rate, kg s^−1^
*A*: Area, m^2^
*H*: Reservoir height, m
*K*: Reservoir permeability, m^2^
*α*: Thermal diffusivity, m s^−2^.

### Subscripts

*γ*: Carbon dioxide
*ε*: Water
*g*: Fluid mixture
*R*: Reservoir
*m*: Rock
*h*: Highest system temperature, at bottom boundary
*c*: Lowest system temperature, at top boundary.
